# Factors Affecting the Implementation Process of Pertussis [Tdap] Immunization in Pregnant Women in an Italian Region: A Qualitative Study

**DOI:** 10.3389/fpubh.2020.00120

**Published:** 2020-04-22

**Authors:** Sara Mazzilli, Lara Tavoschi, Pier Luigi Lopalco

**Affiliations:** Department of Translational Research and New Technologies in Medicine and Surgery, University of Pisa, Pisa, Italy

**Keywords:** Whooping cough, pertussis, Tdap, pregnancy, vaccine implementation, Italy

## Abstract

Whooping cough (pertussis) represents one of the most prevalent vaccine-preventable diseases in Western countries, capable of causing disease in infants, with a high risk of severe complications. To protect new-borns from pertussis, many countries have introduced the acellular pertussis adult vaccine in combination with tetanus and diphtheria toxoids for women in the third trimester of pregnancy. Thanks to the approval of the new National Immunization Prevention Plan 2017–2019: Italy is among those countries. The Italian health-care system is a regionally based National Health Service, therefore, regions organize and implement new vaccination strategy on their own. This study focuses on Tuscany's experience in implementing the maternal pertussis vaccination. The present study had a qualitative design: we obtained information about the implementation process through interviews with relevant stakeholders involved in the planning or implementation of vaccination programme at different levels. We noticed heterogeneous implementation's status between Tuscan Health Care Departments. The most frequently reported barriers influencing the success of the implementation process of this prevention strategy included: lack of accountability, lack of enabling instruments, financial constraints, logistics barriers, training deficiencies, attitudes of health care workers, and lack of communication experts. The implementation of new vaccination programs is complex and challenging. Often the importance of education and information activities targeting health professionals are underestimated and underfunded. These elements would need to be carefully considered and adequate provisions should be made to address them when designing and implementing effective vaccine interventions.

## Introduction

Whooping cough represents one of the most prevalent vaccine-preventable diseases in Western countries (1). Unfortunately, pertussis infection may result in severe disease in infants, with high risk of complications, including death (2). Several scientific studies have shown that after the acellular pertussis adult vaccine in combination with tetanus and diphtheria toxoids (Tdap) administration in the third trimester of gestation pregnant women produce high concentrations of antibodies against pertussis antigens. Through transplacental transfer, anti-pertussis antibodies pass to the fetus, who is protected at the time of birth and during the first months of life (3, 4). In response to the rise of whooping cough cases that was observed in the last decades, since 2012, starting with the United Kingdom and followed by other European countries (Belgium, Spain, France etc.) the Tdap vaccine for pregnant women has been introduced (5–8).

In 2017, Italy approved the new National Immunization Prevention Plan (PNPV) 2017–19. Among new vaccination strategies introduced, there is also the Tdap vaccination for expectant mothers (9). Despite this, data about coverage of pertussis vaccination in pregnant mothers in Italy are still missing.

The Italian health-care system is a regionally based National Health Service (NHS). The system is organized into three levels: national, regional and local. The national level is responsible for establishing the general objectives and fundamental principles of the NHS. The 19 regions and two Autonomous Provinces (R&AP) are responsible for organizing and delivering health care. Within each region, at the local level, local health authorities (LHA) deliver public health services, community health services and primary care directly, and secondary and specialist care directly or through either public hospitals or accredited private providers. The geographical area covered by each LHA is further divided into Health Care Districts (HCD), that are responsible for the provision of public health and primary care services. Each HCD is set to have a coverage population of approximately 60,000 inhabitants (10). In this scenario, through the PNPV, the Ministry of Health has taken the lead in health-care prevention and vaccination planning. Then, the R&AP are in charge of organizing and implementing the new vaccination strategy at the local level based on that document. The introduction of new vaccination and related targets, such as maternal immunization program, posed new challenges to the R&AP Health Authorities (9), even if the strategy has been proven to be safe and effective (11). First, although maternal immunization is an established strategy in many developing countries thanks to efforts to eliminate maternal and neonatal tetanus, it is a relatively new concept in resource-rich countries; second, the target population is composed of healthy adults who may be scarcely aware of the need for and benefit of immunizations; third, there is a general reluctance among pregnant women to take any medications, including vaccines, during pregnancy; fourth, there is the anxiety that adverse pregnancy outcome may be erroneously associated with vaccination, a licit concern given the natural rate of pregnancy loss (12, 13). Finally, there may be logistical and financial barriers; for instance, pregnant women would need to attend vaccination centers as health care professionals (HCPs) who follow them in the ante-natal care (ANC) do not routinely perform immunizations. Focus on policy content and targets of health reform may divert attention from understanding the processes that explain why desired policy outcomes fail to emerge (14). While several studies have explored the level of pertussis maternal immunization knowledge and attitudes among health care professionals and pregnant women (15–18), the perspectives of policy makers and workers who have managerial roles in the health care services have rarely been investigated. However, their actions are extremely important in order to develop efficient maternal vaccination strategies and achieve good vaccination coverages.

This study aims to focus on Tuscany's experience in implementing pertussis vaccine for expectant mothers 1 years after the PNPV has been approved. Between May 29th and September 16th 2018, we performed semi-structured telephone interviews with relevant stakeholder to gain insights into the implementation of maternal pertussis vaccination and identify potential barriers and lessons learnt that may be relevant to facilitate the implementation of prevention policies in other regions in Italy and in other European countries.

## Materials and Methods

### Study Settings

**Figure 1:**
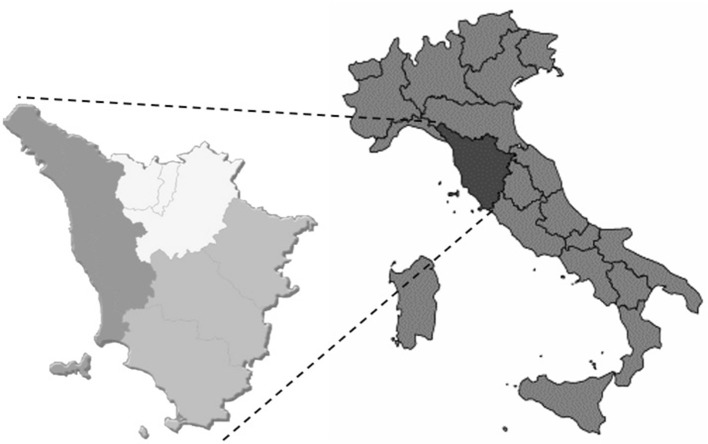
Tuscany's local health authorities. Central Tuscany LHA; South-East Tuscany LHA; North-West Tuscany LHA.

Tuscany is a central Italian region of about 3.7 million inhabitants that, since the January 1st, 2016, is divided into three LHAs: North-West Tuscany LHA, South-East Tuscany LHA and Central Tuscany LHA (Figure 1), that in turn are divided in 26 HCDs. Before 2016, the 26 HCDs were not clustered under LHAs, they acted independently from one another. However, nowadays as in the past, at the district level the immunization delivery is a responsibility of prevention departments. Before the approval of the PNPV, Tuscany prevention departments followed the Tuscany region immunization schedule (TRIS) approved in 2015. According to the TRIS, during pregnancy, women should have verified the vaccination status for Tdap, and if needed, a booster dose should have been performed in the last trimester of gestation (19). However, in the TRIS it was not well-specified when this prevention strategy would be needed; thus, before the introduction of PNPV, we assumed very few pregnant women in Tuscany were vaccinated against pertussis.

### Study Design

This study had a qualitative design and was based on semi-structured telephone interviews.

### Sampling

In order to obtain information about the implementation process we selected stakeholders defined as: policy makers and workers having managerial roles in the health care services who had active role in the planning or implementation of vaccination programme at national, regional, or local level. They have the potential to provide rich, relevant and diverse data pertinent to the study objective. These stakeholders included members of the national and regional committee for vaccination and directors of prevention departments (Table 1). Initially we contacted by email 5 purposively selected stakeholders among our acquaintances. Next, we followed a snowball sampling approach contacting the interviewees' suggestion who met the selection criteria. In total, we carried out 11 in-depth interviews.

**Table 1:** List of stakeholders interviewed.

**Level**	**Respondent**	**Interviewed/contacted by email**	**Respondent code**
National	Italian Ministry of health	1/3	(#1)
	National Commission for Vaccinations	1/1	(#2)
Regional	Prevention department, Regional Health System	1/1	(#3)
Local	Vaccination Responsible at the LHAs level Prevention Department Responsible at the HCD level	0/2 8/13	(#4) (#5) (#6) (#7) (#8) (#9) (#10) (#11)
Total		11/20	

### Data Collection Methods

The interviews were carried out between May 29th and September 16th, 2018. The interviewer had previous experience in conducting expert interviews. We e-mailed purposive selected participants to explain the aims of the research and to book a telephone appointment for the interview. A second mail was sent to the respondents who did not answered to the first mail; in case also the second email went unanswered, when the telephone number of the potential respondent was available on public websites a call was made to ask him/her to participate in the study. The interviews were carried out at a convenient time chosen by the respondent, were conducted in Italian, and lasted 20 min on average. We used a pretested semi-structured Interview Guide (Supplementary Material) to gain insights into the implementation of pertussis vaccination in pregnant women happening as consequence of the approval of PNPV. The guide sought information on issues including the Tdap implementation status, the implementation activities, the logistic of the vaccine distribution and problems emerging during the implementation process. We recorded each interview once informed consent had been sought and obtained. At the end of each interview, we transcribed the recorded conversation in Italian. We anonymized the respondents. The interviews were held to the point of saturation.

### Data Analysis

The researchers carried out a framework analysis in four steps: familiarization, indexing/coding, charting, and mapping/interpretation. First, we familiarized ourselves with the data collected by studying the transcripts. This helped us to gain an overview of the body of material gathered and to become aware of key ideas as well as recurrent themes. Emerging findings were presented for discussion at team meetings, which facilitated further interpretation and refinement of themes and concepts. Our next step was to identify portions of the data that corresponded to a particular theme (indexing or coding). Looking through the data we identified a number of themes, which we then organized under different categories. Thereafter, with the use of NVivo 12 Pro, we lifted the indexed data from its original textual context and put these data in charts that organized the themes into categories and sub-categories. As themes emerged these were indexed and compared with themes from subsequent interviews. We did an interpretation which involved the analysis of the key characteristics as laid out in the charts. Lastly, the various trends, opinions, and perspectives of the researchers together with their identified related verbatim quotes were used to produce a narrative and summary of the findings written in English. The verbatim quotes included in this manuscript were translated at this point. All the authors agreed with the translation presented.

### Ethics Approval and Consent to Participate

During the study planning period we checked the requirements of the competent Ethics Committee for the University of Pisa (https://www.unipi.it/index.php/etica-nella-ricerca/itemlist/category/1322-comitato-bioetico-dell-universita-di-pisa). The guidelines for seeking ethics approval clearly define the studies for which the ethics committee review and approval is required. Our study did not foresee the involvement of patients, medical interventions of any sort, or the conduct of experiments on animals. Hence, according to the competent Ethics Committee it did not require ethics approval. The guidelines are publicly available here: https://alboufficiale.unipi.it/wp-content/uploads/2017/12/regolamento.pdf.

We did comply with the requirements of informed consent and anonymization: we obtained a recorded verbal consent from each respondent following explanation of the study's aims and objectives. We performed interviews using a smartphone. During each session, permission was also sought and obtained from participants before setting the telephone in speakerphone and switching on the audio recorder. All participants were anonymized and identified with alphanumeric/numeric code prior to data analysis.

## Results

**Figure 2:**
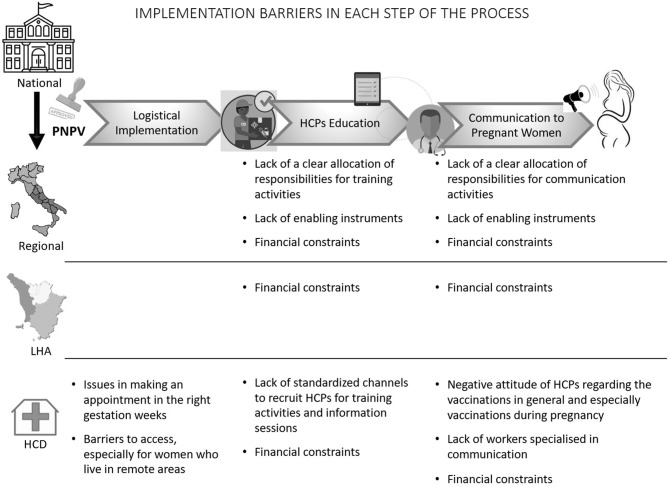
Implementation barriers in each step of the process.

Among the 20 stakeholder we contacted by email 11 were available for the telephone interview, three of the remaining recommended we interview their colleagues who were reportedly more experienced in vaccination for pregnant women, two were interested but it was not possible to schedule an appointment, four did not respond to our emails or calls. In this section we presented results divided in two subsections: in the first, we describe the status of the implementation process in Tuscany. In the second, we grouped the factors influencing the implementation into two sub-categories: health system factors and human resource factors (Figure 2).

### Status of Implementation Process

According to PNPV, regions are in charge of implementing new vaccination strategies contained in the document. Therefore, Tuscany region has recognized the plan with an implementation act sent to all the HCDs; moreover, the PNPV has been divulged to the prevention departments which, according to the regional responsible for the prevention, are in charge of contacting the stakeholder responsible for implementing the plan. Tuscany region also designed communication and information plans to promote the PNPV, however, the pertussis vaccine for pregnant women was hardly mentioned. Despite the approval of the PNPV the TRIS was not updated after 2015. Nevertheless, three of the directors of prevention departments interviewed, said that they started the implementation of pertussis vaccination for pregnant women before the PNPV's approval. Other interviewees provided different responses: six stating that the delivery of pertussis vaccination of pregnant women, in their HCD, was implemented after the issuance of PNPV, while the remaining two indicated that in their area the implementation of this prevention strategy was going to be discussed in the near future with other prevention departments clustered in the same LHA.

In any case, the PNPV has been an important instrument to promote the Tdap vaccination during pregnancy, even where implementation began before 2017:

“*R: after the approval of the PNPV, in your area, have you seen changes regarding the implementation of Tdap vaccination for pregnant women?*

*I: yes, there were positive changes, […] here the health care professionals are worried about their responsibilities, but now that also an official document like the PNPV discussed it* (pertussis vaccination in pregnancy) *we can overcome oppositions more easily” [#8]*

The lack of guidelines given by the region caused not only differences in progress of implementation activities between HCDs so that the status of the implementation depends almost exclusively on the decisions of responsible of prevention departments, but also differences in the way in which this prevention strategy has been put into action. In the HCDs where the implementation has started, women are informed about the importance of this vaccination with different approaches:

“*I: the gynecologist is the person who gives to the woman the recommendation, but we also prepared a leaflet which is given to the expectant mother by the obstetrician. Both professionals are important to the mother to be” [#5]*

“*I: as the prenatal screening is carried out for all unborn children, when the woman goes to do this exam, information about the importance of vaccination during pregnancy is given through a leaflet” [#6]*

“*I: during the prenatal courses we have introduced 2-3 hours with pediatrics: in this occasion, we give information on pregnant women vaccination. We perform this activity once a month.” [#4]*

Despite these differences in the pathway used to inform women about the Tdap vaccine, in all the HCDs the Tdap vaccine for expectant mothers are provided by the vaccination center of prevention department. Only one of the respondents mentioned that in his HCD they were willing to vaccinate pregnant women inside the hospital gynecology unit: in this way gynecologists and obstetricians could directly vaccinate mothers to be.

### Health System Factors

#### Accountability

All the respondents agree to the fact that the vaccination centers are responsible for the delivery of the vaccination; however, it is more difficult to understand from the interviews who is accountable for general practitioners' (GPs), gynecologists' and midwives' education and for the communication to the public. For instance, with respect of the HCPs' training the regional responsible for the prevention said:

“*I: the Region makes a training plan for general practitioners, but we have never managed to do a regional course on vaccination, because they are oriented on other subjects. […] We did not follow the gynecologists directly, HCDs should organize courses” [#3]*

And:

“*I: this is an activity* (organizing the implementation of Tdap vaccination for pregnant women) *under the responsibility of the prevention departments, so they have to organize training campaigns on the topic at local level” [#3]*

While the directors of local prevention units have different opinion about their role and their responsibility for the training of HCPs, four believe that the training promotion should start from the region and courses should be organized at the LHA level; two think that the training on these topics should be given through professionals' organizations, whilst the other two recognize their accountability to organize training on vaccination for HCPs.

There is no clear view on what health institution should be in charge of the communication with the public. However, four of the respondents believed that communication campaigns and materials from the region would have more credibility and authority.

#### Lack of Enabling Instruments

The respondents at national level and five of the respondents at local level highlighted the fact that regions should produce more instruments to facilitate the implementation of this prevention strategy.

One of the most mentioned tools was the ANC booklet which is given to each pregnant woman to guide ante-natal care and help navigating the health care services. Respondents at the local level suggested that the ANC booklet should be updated by the region and information on vaccination during the last trimester of pregnancy should be introduced.

As the ANC booklet is consulted not just by mothers to be, but also by GPs, gynecologists and other HCPs, prevention departments directors see this booklet as a good instrument to promote vaccination. Another instrument that prevention departments directors consider useful are informative leaflets, with all the relevant information about Tdap vaccination, made by the region and suitable to be shared with all the stakeholders, pregnant women and HCPs.

#### Financial Constraints

While all respondents claim that there is adequate funding to offer the vaccination service to pregnant women for free, seven respondents complained that inappropriate funding was one of the main barriers to the organization of educational events and to the implementation of the vaccination communication interventions.

Respondents at local level also complained that they have no founds to dedicate to training and communication activities:

“*I: the training activities are based on people of good will who are involved in them, but they are not remunerated for them” [#9]*

#### Logistics Barriers

Four of the respondents at local level highlighted that because the pregnant women should get the vaccine over a specific period of the pregnancy, they can have difficulties in making an appointment in the right gestation weeks, because too often centers of vaccination have long waitlist. Moreover, a respondent mentioned the fact that vaccination centers are not dislocated in each town: if a woman lives in a remote area, it can take her a long time to arrive at the vaccination center.

### Human Resource Factors

#### Training Deficiencies

The lack of knowledge about this prevention strategy of GPs, gynecologists and midwives is one of the most commonly mentioned barriers to the implementations of the Tdap vaccination for pregnant women. Moreover, two of the respondents have pointed out that the members of these professionals' categories worked in many different structures: hospitals, private ambulatories, clinics, and there is no standardized channel through which they can be contacted to provide them with information and training about new practices. One respondent was also of the view that health workers had poor communication skills and were not able to properly communicate the purpose of the pertussis vaccination. Therefore, for the majority of the respondents, to organize training for HCPs is fundamental to achieve a good implementation of this prevention practice.

#### Attitudes of Health Care Workers

Most of the respondents have highlighted the negative attitude of midwives regarding the vaccinations in general and especially vaccinations during pregnancy. Indeed, the respondents stated that midwives tend to see the pregnancy as a natural process in which the medicine should interfere as little as possible.

“*I: the midwives generally advice against vaccinations: we tried many times to do something for this category, but they have a naturalistic idea of diseases” [#3]*

Moreover, some respondents highlighted that even some gynecologists are hesitant about the effective need of pertussis vaccination during pregnancy:

“*I: not all the gynecologists are inclined to recommend the vaccine, not all of them perceive the value of the Tdap vaccination administered during pregnancy to prevent the pertussis infection for the unborn child. There is a problem with HCPs who have not familiarity with vaccinations. Gynecologists, except for papilloma virus vaccination, have no experience with vaccinations.” [#8]*

#### Lack of Workers Specialized in Communication

The informative leaflet used in two of the responders' HCDs to promote Tdap vaccine for pregnant women were designed by the personnel of the vaccination departments, who do not have any competences in communication. This make us understand that at the local level there is a lack of workers expert in the communication field that could take responsibility for the communication campaigns.

## Discussion

To the best of our knowledge this is the first study exploring the implementation process of Tdap maternal vaccination in Italy. This paper describes the status and outlines some of the most relevant factors influencing the process which may be useful when planning and implementing Tdap maternal immunization programmes in other Italian and international settings. In our study, we noticed heterogeneous implementation statuses, even between Tuscan HCDs: in some of them the implementation process has not even started yet. According to the WHO Department of Immunization, Vaccines and Biologicals, once a decision to introduce a vaccine has been made, an immunization programme manager should develop a new vaccine “introduction” plan as well as detailed “implementation” plans with specific timelines (20). Through TRIS, the Tuscany region has entrusted HCDs to put in action the Tdap vaccination to pregnant women, but, unfortunately, the immunization schedule does not contain any detailed introduction and implementation plans for Tdap maternal immunization. This absence in the TRIS may be the reason for the observed heterogeneity in levels and modes of implementation. Even if our study was focused on Tuscany, the fact that the MoH in August 2018, published an implementation act entitled “recommended vaccination for women in childbearing years and for pregnant women.” could suggest that the implementation of vaccinations during pregnancy was sub-optimal also in other Italian regions. Indeed, in this implementing act the MoH not only reiterates the importance of vaccinations as a prevention instrument to protect mothers and their unborn children, but also urges R&AP to include vaccinations between the actions provided for maternal and child health and to promote, with appropriate communication campaigns, vaccinations for women in childbearing years, for pregnant women and for women in the puerperium (21).

According to our findings and in line with existing literature, one of the key elements in the successful introduction of a vaccine is the high-quality training of all health workers involved in the process, about the new vaccine and the disease it prevents (20). Moreover, communication is particularly necessary to achieve high vaccination coverage in hard-to-reach populations like pregnant women (22). In our study we found that some of the local respondents were not planning any training and communication activities: they considered LHAs or the regional prevention department responsible for that. Those who have organized educational and communication activities still claimed the lack of enabling instruments, to promote the vaccine, both to HCPs and to pregnant women. In any case, at the district level there was hardly any financial or human resource to dedicate to the development and implementation of communication and training (Table 2). These findings confirm those obtained by Signorelli et al., who studied the introduction of meningococcal serogroup B vaccine in Italian regional immunization schedules: they found that the lack of health education and communication activities and the economic sustainability in a context of deprived resources are main barriers to the implementation process (23). Moreover, the results of Mita et al., who studied rotavirus vaccine implementation after the approval of the PNPV, are consistent with our findings (24). Some of our respondents suggested that the ANC booklet should be updated with information about immunizations needed in the antenatal period, and this had also been suggested in other studies (12). According to the respondents, one of the most common barriers to implementation is the lack of knowledge among HCPs. Indeed, according with Winslade et al. study, in most of the cases, poor uptake of vaccines by pregnant women is not due to active refusals but to a lack of recommendation by HCPs (25). The support for new vaccination programs by HCPs is correlated with self-assessed level of knowledge (26). To mitigate this problem, a possible solution consists in the provision of auditable educational training to HCPs contributing to ANC services such as GPs, gynecologists and midwives, to improve their confidence and inclination to discuss vaccination with expectant mothers. A second critical issue, emphasized by many respondents, is the reported skeptical attitude toward vaccinations among midwives and gynecologists in their district: this is consistent with findings in the literature (27, 28). For this reason, in order to understand why HCPs have this attitude, university courses designed for midwives and gynecologists should be revised. Surprisingly, education on vaccines and vaccination is sub-optimal in the medical curricula in most European countries (29). Hence, efforts should be made in order to incorporate more extensive training on vaccinology for future generations of midwives and gynecologists.

**Table 2:** Issues and potential solutions to better support the implementation of maternal Tdap vaccination.

**Health system issues**	**Suggested recommendations to support implementation**
Accountability	Develop a detailed “implementation” plan with clear allocation of responsibilities within and across institutions
Lack of enabling instruments	Update the booklet for the pregnancy Develop and distribute educational and informational materials through prevention departments
Financial constraints	Provide a regular source of funding at the local level for routine immunization communication and training activities
Logistics barriers	Ensure preferential referral pathways for pregnant women to access vaccination centers
Training deficiencies	Establish a system to provide training activities to the HCPs offering/recommending the vaccine to pregnant women
Attitudes of health care workers	Revise university curricula Organize extensive training on immunization
Lack of workers specialized in communication	Hire communication experts at the local level

The main limitation of this study is the high number of experts that did not answer to our emails or calls and was impossible to reach. The responders may be the most informed and most concerned one about the topic of our research and they may not be representative of all the possible stakeholders.

In conclusion, in this study we described the status of implementation of pertussis vaccine for pregnant women in Tuscany region, following the approval of the PNPV. Moreover, we outlined some of the key issues influencing the success of the implementation process of this prevention strategy. In summary, the implementation of new vaccination programs is complex and challenging in many respects: to achieve the objectives set in the PNPV is important to understand operational and implementation challenges that may occur during the implementation process. Often the importance of education and information activities are underestimated and underfunded. Support for new vaccination programs by health professionals should not be taken for granted. These elements would need to be carefully considered and adequate provisions made when designing and implementing vaccine interventions.

## Data Availability Statement

The datasets generated for this study are available on request to the corresponding author.

## Ethics Statement

Ethical review and approval was not required for the study on human participants in accordance with the local legislation and institutional requirements. Written informed consent for participation was not required for this study in accordance with the national legislation and the institutional requirements. Written informed consent was not obtained from the individual(s) for the publication of any potentially identifiable images or data included in this article.

## Author Contributions

SM collected the data. SM, LT, and PL analyzed the data. SM wrote the first draft of the article with input from LT and PL. All of the authors read and contributed to the manuscript and agree with the material presented, and developed the research protocol.

## Conflict of Interest

The authors declare that the research was conducted in the absence of any commercial or financial relationships that could be construed as a potential conflict of interest.
