# Supplementary material for: Factors Affecting the Implementation Process of Pertussis [Tdap] Immunization in Pregnant Women in an Italian Region: A Qualitative Study
Source: Front Public Health. 2020 Apr 22;8:120. doi: 10.3389/fpubh.2020.00120 (PMC7212430; doi:10.3389/fpubh.2020.00120)
Supplement: Supplementary file 1 [file Data_Sheet_1.docx]

INTERVIEW GUIDE

This research project has the aim to explore Tuscany’s experience in implementing the maternal pertussis vaccination mentioned in the Italian PNPV.

Within this research I plan to collect information interviewing policy makers and workers who have managerial roles in the health care services, that is why I ask you to do this interview.

I will use what you will say for the research purpose. Do you agree?

I would need to record this interview for the data analysis purpose. Do you agree?

All your personal data will be anonymized and there will not be the way to trace back to you.

The PNPV it is written: “Vaccination during pregnancy against diphtheria, tetanus, pertussis is of great importance. In fact, whooping cough contracted in the first few months of life can be very serious or even fatal […]. For these reasons, vaccinating the mother in the last weeks of pregnancy allows the passive transfer of antibodies capable of immunizing the newborn until the development of an active child's vaccination protection. The vaccine has proven safety for both the pregnant woman and the fetus.” According to your opinion, what practical implications will these claims have on the pertussis vaccine implementation for pregnant women?

- Negative response (eg. “it will not have implications”)

Why are you making this statement? (What did not work in the implementation process? What are the reasons? What are the main barriers hampering its implementation?)

How should it be implemented?

Details:

• time

• regional and territorial responsibility

• who, where and how the vaccine should be administered

• recommendation

• training of health professionals

• information for pregnant women

• cost

- Positive response

How has it been / will be implemented?

Details:

• time

• regional and territorial responsibility

• who, where and how the vaccine should be administered

• recommendation

• training of health professionals

• information for pregnant women

• cost

What are / would be the limits of this type of implementation?

What are / would be the main difficulties / barriers for implementation on a national / regional scale?

- 2. What information could be useful to policy makers in order to implement the vaccination strategy efficiently?
- 3. Could you suggest to me someone who is experienced in this field (or who is in charge of the of vaccinations implementation) and could help me with my research?
